# Differential regulation of RhoA-mediated signaling by the TPα and TPβ isoforms of the human thromboxane A2 receptor: Independent modulation of TPα signaling by prostacyclin and nitric oxide

**DOI:** 10.1016/j.cellsig.2008.04.006

**Published:** 2008-08

**Authors:** Katarina Wikström, David J. Kavanagh, Helen M. Reid, B. Therese Kinsella

**Affiliations:** UCD School of Biomolecular and Biomedical Science, UCD Conway Institute of Biomolecular and Biomedical Research, University College Dublin, Belfield, Dublin 4, Ireland

**Keywords:** GPCR, G protein-coupled receptor, HA, hemagglutinin, HEK, human embryonic kidney, IP, prostacyclin receptor, IP_3_, inositol 1, 4, 5-trisphosphate, NO, nitric oxide, eNOS, endothelial nitric oxide synthase, PAGE, polyacrylamide gel electrophoresis, PG, prostaglandin, PK, protein kinase, PL, phospholipase, sGC, soluble guanylyl cyclase, SIN-1, 3-morpholinosydnonimine, HCl, TP, TXA_2_ receptor, TX, thromboxane, VSM, vascular smooth muscle., Thromboxane receptor, RhoA, Nitric oxide, Prostacyclin, Protein kinase, Heterologous desensitization

## Abstract

In humans, thromboxane (TX) A_2_ signals through the TPα and TPβ isoforms of the TXA_2_ receptor that exhibit common and distinct roles. For example, Gq/phospholipase (PL)Cβ signaling by TPα is directly inhibited by the vasodilators prostacyclin and nitric oxide (NO) whereas that signaling by TPβ is unaffected. Herein, we investigated whether TPα and/or TPβ regulate G_12_/Rho activation and whether that signaling might be differentially regulated by prostacyclin and/or NO. Both TPα and TPβ independently regulated RhoA activation and signaling in clonal cells over-expressing TPα or TPβ and in primary human aortic smooth muscle cells (1° AoSMCs). While RhoA-signaling by TPα was directly impaired by prostacyclin and NO through protein kinase (PK)A- and PKG-dependent phosphorylation, respectively, signaling by TPβ was not directly affected by either agent. Collectively, while TPα and TPβ contribute to RhoA activation, our findings support the hypothesis that TPα is involved in the dynamic regulation of haemostasis and vascular tone, such as in response to prostacyclin and NO. Conversely, the role of TPβ in such processes remains unsolved. Data herein provide essential new insights into the physiologic roles of TPα and TPβ and, through studies in AoSMCs, reveal an additional mode of regulation of VSM contractile responses by TXA_2_.

## 1 Introduction

The phosphorylation status of myosin light chain (MLC) of the actomyosin complex plays a central role in regulating the various types of cytoskeletal reorganizations that widely occur within the vasculature including in platelet shape change and aggregation, tonic- or agonist-induced contraction and relaxation of smooth muscle cells (SMCs), cell migration, cell proliferation and stress fibre formation [1]. Many of the physiologic regulators of platelets and vascular smooth muscle (VSM) contraction, including thromboxane (TX) A_2_, thrombin, ADP, prostaglandin (PG) I_2_ or PGD_2_, act through specific G protein coupled receptor (GPCR) -effector systems [1]. While agents such as TXA_2_ and thrombin that promote platelet activation or SMC contraction induce Gq-dependent phospholipase (PL)Cβ activation to evoke calcium (Ca^2+^) -dependent activation of myosin light chain kinase (MLCK) and MLC_20_ phosphorylation [1,2], they may also engage the Ca^2+^-independent pathway involving receptor co-coupling to G_12_ and RhoA signalling [1]. G_12_ members, particularly Gα_13_, activate RGS (regulators of G protein signaling)-box containing members of the Rho guanine nucleotide exchange factor (RhoGEF) family, such as p115 RhoGEF, PDZ RhoGEF or LARG, to activate RhoA and its key effector in this system Rho kinase 1 (also known as p164 ROKα/ROCK2) and 2 (p160 ROKβ/ROCK1), herein termed Rho kinase/ROCK [3–5]. Rho kinases, in turn, phosphorylate, and inactivate, myosin phosphatase, MLC itself and the myosin phosphatase inhibitor CPI-17 resulting in the Ca^2+^-independent increase in overall levels of phosphorylated MLC through a Rho/Rho kinase-mechanism [2,4,5]. Other targets of Rho kinase include its phosphorylation-dependent activation of LIM kinases which, in turn, phosphorylate and inactivate the actin depolymerizing agent cofilin [4]. The central importance of the Ca^2+^-independent mechanism of contraction within the vasculature has been highlighted through findings that disorders of the Rho/Rho kinase pathway are major underlying causes of hypertension, vascular spasm and atherosclerosis making Rho kinase an important therapeutic target in the treatment of these diseases [1,2,6].

The prostanoid TXA_2_ plays an essential role within the vasculature inducing a diversity of cellular responses including platelet shape change, secretion and aggregation, VSMC contraction and migration and is widely implicated in a number of cardiovascular disorders including thrombosis, hypertension, vessel remodelling and atherosclerotic progression [7]. As a predominantly Gq/PLCβ-coupled GPCR, the TXA_2_ receptor or TP can induce both Ca^2+^-dependent and G_12/13_-mediated RhoA/Ca^2+^ independent responses platelets and VSMCs [1,8]. For example, platelets from Gα_13_-deficient mice do not undergo RhoA-dependent shape change in response to low levels of TXA_2_ but retain the ability to undergo Gq/Ca^2+^-dependent shape change and aggregation at higher agonist concentrations [9]. Similarly, both Ca^2+^-dependent/PLCβ and Ca^2+^-independent/RhoA mechanisms contribute to TXA_2_-induced contraction in isolated bovine aortic (Ao) SMCs and in VSM tissue from various other species [10–12]. Notably however, in humans, but not in non-primates, TXA_2_ actually signals through two distinct TXA_2_ receptor isoforms termed TPα and TPβ that arise through alternative splicing and differ exclusively in their carboxyl-terminal (C tail) domains [13–15]. Whilst it is currently unknown whether TPα or TPβ independently or indeed differentially modulate RhoA activation and downstream signaling, there is substantial evidence that the TPα and TPβ isoforms can differentially regulate other cellular effectors raising that possibility [16–21].

While both TPα and TPβ are predominantly coupled to Gq/PLCβ activation [22], they can independently regulate other secondary effector systems including opposite regulation of adenylyl cyclase via Gs and Gi, respectively [23]. Additionally, TPα, but not TPβ, couples to PLCδ activation via Gh/tissue transglutaminase [24]. Whereas both TPs are expressed in VSMCs, TPα is the predominant isoform expressed in human platelets [25,26]. Consistent with this, in studies investigating intermolecular cross talk between the pro-aggregatory TXA_2_ and the inhibitory prostanoid prostacyclin (PGI_2_), it was established that Gq/PLCβ coupling and signaling by TPα, but not TPβ, undergoes prostacyclin- induced desensitization mediated through direct cAMP-protein kinase (PK) A phosphorylation of TPα at Ser^329^ within its unique C-tail domain [21,27]. Moreover, Gq/PLCβ signaling by TPα, but not TPβ, is also desensitized by the platelet antagonist /vasodilator nitric oxide (NO), involving direct NO/cGMP-dependent PKG phosphorylation of TPα also within its unique C-tail [20] The implication from these studies is that TPα plays a critical role in vascular haemostasis acting as the major TP target for regulation/inhibition by prostacyclin and NO, such as within the anucleate platelet that predominantly expresses TPα. However, the impact of such direct inhibitory effects of prostacyclin and NO mediated by PKA and PKG, respectively, on signaling by TPα and TPβ through other effector systems, such as through RhoA, is currently unknown but, clearly, any differential regulatory effects by either prostacyclin or NO on such TXA_2_ signaling may have direct clinical implications, for example within human VSMCs that express both TPα and TPβ isoforms. Hence, the aim of the current study was to investigate whether TPα and/or TPβ independently regulate G_12_/Rho activation and signaling and to establish whether that signaling is differentially regulated by the inhibitory prostacyclin/cAMP/PKA and NO/cGMP/PKG systems. These studies provide essential new insights into the physiologic roles of TPα and TPβ and, through studies in primary human aortic smooth muscle cells (1° AoSMCs), reveal an additional mode of regulation of VSM contractile responses by the potent autocoid TXA_2_.

## 2 Materials and methods

### 2.1 Materials

U46619, SQ29,548, BW245C, FK409, FURA2/AM were purchased from Cayman Chemical Company; SIN-1 and Y-27632 from Calbiochem; 3F10 *anti*-HA, 3F10 *anti*-HA-horseradish peroxidase (HRP)-conjugated antibody and chemiluminescence detection kit from Roche; *anti*-RhoA 26C4 (Sc-418), *anti*-phospho-RhoA^Ser188^ (Sc-32954-R), *anti*-Gα_12/13_ H-300 (Sc-28588), *anti*-Gαq C15 (SC-392), HRP-conjugated goat *anti*-mouse (Sc-2005), HRP-conjugated mouse *anti*-goat (Sc-2354) and HRP-conjugated goat *anti*-rabbit (Sc-2004) antibodies from Santa Cruz; Glutathione-Sepharose 4B (GE Healthcare) and FITC conjugated goat *anti*-rabbit antibody from Sigma; *anti*-cofilin (# 3312) and *anti*-phospho-cofilin (phospho^Ser3^, # 3311) were from Cell Signaling; Alexa Fluor® 488 phalloidin (A12379; Excitation / Emission A_495/518 nm_) from Molecular Probes; *anti*-HDJ2 antibody from Neomarkers; Opti-MEM® and Oligofectamine® were from Invitrogen. All oligonucleotides were synthesised by Genosys Biotechnologies; small interfering (si) RNAs by Qiagen. Cicaprost was a gift from Schering AG (Berlin, Germany). pcDNA3.1(+):hGαq^Q209l,D277N^, pCis:Gα_12_^G228A^ and pCis:Gα_13_^G225A^ were from the UMR cDNA Resource Center (Gαq) or from Dr S. Offermanns, University of Heidelberg, Germany.

### 2.2 Cell culture and transfections

Human embryonic kidney (HEK) 293 cells were grown in minimal essential medium (MEM), 10% foetal bovine serum (FBS). HEK.TPα, HEK.TPβ, HEK.TPα^S329A^ HEK.TPα^S331A^ and HEK.TPα^S329,331A^ cell lines stably over-expressing hemagglutinin (HA) -tagged forms of TPα, TPβ, TPα^S329A^,TPα^S331A^ and TPα^S329,331A^ respectively, have been previously described [20,21]. For transfections, HEK 293 cell lines were routinely plated 48 h previously at ~ 2 × 10^6^ cells/10 cm dish in 8 ml media and co-transfected with 10 μg of pADVA and 25 μg of pCMV-based mammalian expression vector using the calcium phosphate/DNA co-precipitation procedure [20].

Primary (1°) human aortic smooth muscle cells (1° h.AoSMCs) were purchased from Cascade Biologics (C-007-5C) and routinely grown in Smooth Muscle Cell Growth Medium 2 (Promocell GMBH, C-22062) supplemented with 0.5 ng/ml epidermal growth factor, 2 ng/ml basic Fibroblast growth factor, 5 μg/ml insulin, 5% FBS.

### 2.3 Calcium measurements

Measurements of intracellular calcium ([Ca^2+^]_*i*_) mobilization were carried out in FURA2/AM preloaded HEK 293 cell lines transiently co-transfected with pCMV:Gαq and pADVA some 48 h previously, as described [20]. Cells were stimulated with 1 µM U46619, 1 µM Cicaprost, 1 μM BW245C, 5 μM SIN-1 or 10 μM FK409, unless otherwise specified. Data (Supplemental Figs. 1 and 2) are representative of 3–4 independent experiments and calculated as changes in intracellular Ca^2+^ mobilized (Δ[Ca^2+^]_*i*_ (nM)) as a function of time (seconds, s) following ligand stimulation.

### 2.4 Determination of RhoA activation and cofilin phosphorylation

Activated cellular Rho was determined by interaction with a purified glutathione-S-transferase: rhotekin Rho-binding domain (GST-RBD) fusion protein immobilized on Glutathione-Sepharose 4B resin [28]. Preparation of the GST-RBD protein was carried out as previously reported [28]. For the ‘Rho-pulldown assay’, in brief, HEK.TPα, HEK.TPβ, HEK.TPα^S329A^ HEK.TPα^S331A^, HEK.TPα^S329,331A^ or 1° h.AoSMCs cells were plated some 48 h previously in complete growth medium onto 10-cm dishes to achieve ~ 70% confluency; cells were then serum starved for 5 h or 20 h (1° h.AoSMCs cells) in growth media containing 0.1% FBS before stimulation for 0–30 min with 0–10 μM U46619, as indicated in specific figure legends. To assess the effect of prostacyclin, nitric oxide (NO) or PGD_2_ on TP-mediated Rho signaling, cells were pre-incubated for 10 min with either 0.01–10 μM Cicaprost; 0.05–50 μM SIN-1; 10 μM FK409 or 1 μM BW245C before stimulation with U46619 (typically 0.1 μM for 10 min). As controls, cells were incubated with an equivalent volume of the drug vehicle, agonist or inhibitor in 0.01% ethanol in HBS (modified Ca^2+^/Mg^2+^-free Hank's buffered salt solution) for equivalent incubation times.

Thereafter, cells were lysed in 800 μl Lysis Buffer (125 mM HEPES, pH 7.5, 750 mM NaCl, 5 mM EDTA, 5% NP-40, 10% glycerol, 50 mM MgCl_2_, and 10 μg/ml each of leupeptin and aprotinin; [29]) and aliquots (600 μl) were subjected to pulldown using Glutathione-Sepharose 4B beads preloaded with 30 μg GST-RBD, essentially as previously described [28]. Following washing, precipitated GTP-bound RhoA was subjected to SDS-PAGE on 12.5% acrylamide gels and immunoblotted with *anti*-RhoA monoclonal antibody (Sc-418), followed by chemiluminescence detection [21]. In parallel, to confirm equivalent RhoA protein expression in the cell lysates and uniform protein loading, aliquots of whole cell lysates (typically 10 μl, corresponding to 1.25% of total cell lysate) were directly immunoblotted with the *anti*-RhoA antibody and/or with the anti-HDJ2 antibody. Similarly, to assess U46619-mediated cofilin phosphorylation and activation, aliquots of whole cells lysates (typically 10 μl, corresponding to 1.25% of total cell lysate) were first immunoblotted with *anti*-phospho^Ser3^ cofilin antibody; thereafter, phospho-cofilin blots were stripped and rescreened versus *anti*-cofilin antibody to normalise for total cofilin protein expression and/or with the anti-HDJ2 antibody to confirm uniform protein loading in each of the assays. All images of RhoA expression/pulldown or cofilin phosphorylation and/or expression were captured using Adobe Photoshop (V6), where band width and intensity was quantified and represented as fold increases relative to basal levels. To account for biological variations in basal activation levels, experiments were normalised to within a comparable range based on measurements from more then 20 individual experiments for each cell type.

### 2.5 F-actin staining

HEK 293 cell lines or 1° h.AoSMCs, grown on coverslips for 3 days to achieve approximately 50% confluency, were serum-starved for 2 h in growth media containing 0.1% FBS, prior to stimulation with U46619 (0–1 μM; typically 10 nM U46619). To assess the role of prostacyclin or NO, cells were pre-incubated for 10 min with either 0.01–10 μM Cicaprost or 0.05–50 μM SIN-1 before stimulation with U46619 (typically 10 nM for 10 min). F-actin polymerization was stained by the addition of Alexa Fluor® 488 phalloidin essentially as described by the supplier (Molecular Probes) and slides were imaged using an Axioplan 2 Imaging Universal Microscope.

### 2.6 Disruption of TPa and TPβ expression by small interfering (si) RNA

For RNA_*interference*_ (RNA_*i*_) experiments, HEK 293 cell lines (HEK.TPα, HEK. TPβ and, as controls, HEK293 cells) or 1° h.AoSMCs were plated at ~ 2.5 × 10^5^ cells /35-mm plate and were allowed to attach for 24 h, achieving ~ 30 % confluency. Thereafter, cells were washed twice with serum-free Opti-MEM® and transfected for 4 h at 37 °C with 0.2 μM TPα siRNA (siRNA_TPα_; a 50:50 mixture of two individual 19 bp siRNAs duplexes corresponding to nucleotides 2003–2021 and 2380–2398 of GenBank accession D38081, respectively) or 0.2 μM TPβ siRNA (siRNA_TPβ_; a 50:50 mixture of two individual 19 bp siRNAs duplexes corresponding to nucleotides 1966–1974 + 2634–2644 and 1970–1974 + 2634–2647 of GenBank accession D38081, respectively) or 0.2 μM Lamin A/C siRNA (5'-CUGGACUUCCAGAAGAACAtt) using Oligofectamine® (3 μl/well) in Opti-MEM® (1 ml /well). Thereafter, 1 ml pre-warmed complete media supplemented with 30% FCS was added per well and cells were harvested following incubation at 0, 24, 48, and 72 h. As additional controls, HEK.TPα cells were treated according to the latter conditions but using TPβ siRNAs and *vice versa*.

To confirm the efficacy of the siRNAs to disrupt TPα or TPβ expression, HEK 293 cell lines were harvested and subject to SDS-PAGE (25–50 μg/lane on 12.5% gels) followed by electroblotting onto PVDF membranes (Roche). Membranes were initially screened versus the *anti*-HA (3F10) antibody and, following stripping, were rescreened versus *anti*-HDJ antibody to confirm uniform protein loading. Similarly, 1° h.AoSMCs were screened, under permabilising conditions, by indirect immunofluorescence microscopy for TPα and TPβ expression using affinity purified isoform specific rabbit *anti*-TPα (3 μg/ml) and *anti*-TPβ (3 μg/ml) antibodies [30] incorporating tyramide signal amplification (TSA system; Invitrogen), used as per the manufacturer's instructions. In brief, following incubation with the primary antibodies, 1° h.AoSMCs were incubated with biotinylated goat anti-rabbit (1 in 500 dilution), followed by streptavidin HRP (1 in 2000). Signal amplification was facilitated by incubating the HRP labeled cells with biotinylated tyramide for 10 min at room temperature. Thereafter, 1° h.AoSMCs were incubated with streptavidin FITC (1 in 1000 dilution) and counterstained with propidium iodide (20 μg/ml), prior to mounting and imaging using a Zeiss fluorescence microscope coupled with AxioVision Software (V 4.4).

Thereafter, having optimised the conditions for effective RNA_*i*_ disruption of TPα or TPβ expression in respective HEK 293 lines and in 1° h.AoSMCs, experiments was scaled up 8.2-fold (2 × 10^6^ cells on 10-cm dishes) and functional disruption was assessed through Rho pulldown assays or cofilin phosphorylation, as previously outlined herein.

### 2.7 Data analyses

Radioligand binding data was analyzed using GraphPad Prism V3.0 to determine the K*d* and *B*_max_ values. Statistical analyses were carried out using the unpaired Student's *t* test using the Statworks Analysis Package. *p-*values of less than or equal to 0.05 were considered to indicate a statistically significant difference. Throughout the figures, ⁎ < 0.05, ⁎⁎< 0.01, ⁎⁎⁎< 0.001.

## 3 Results

### 3.1 TPα and TPβ isoforms independently regulate the Gq/PLCβ and G_12_/Rho signaling systems

Whilst a range of studies have investigated Gq/PLCβ-mediated signaling by both the TPα and TPβ isoforms of the TXA_2_ receptor (TP) expressed in human tissues, to our knowledge, no such study has investigated the propensities or relative abilities of the individual TPα or TPβ isoforms to activate and/or regulate Rho-mediated signaling. Hence, herein we investigated TPα and TPβ-mediated Rho signaling in response to the TXA_2_ mimetic U46619 in clonal HEK 293 cell lines that stably over-express either TPα (HEK.TPα cells) or TPβ (HEK.TPβ cells). Throughout these studies, TPα/TPβ-mediated Gq/PLCβ-dependent [Ca^2+^]_*i*_ mobilization was monitored as a comparative reference.

Consistent with previous reports [20,21], both TPα and TPβ expressed in HEK.TPα cells and HEK.TPβ cells, respectively, showed similar concentration-dependent mobilization of [Ca^2+^]_*i*_ in response to U46619 stimulation, with maximal responses generated using 1 μM U46619 (Fig. 1; Supplemental Data). Moreover, both TPα and TPβ also mediated rapid RhoA activation in HEK.TPα and HEK.TPβ cells in response to U46619 stimulation while no such activation was observed in the vehicle-treated cells or in the control non-transfected HEK 293 cells in the presence of U46619 (Fig. 1A). From concentration-response studies, 10–100 nM U46619 was required for maximal RhoA activation by both TPα and TPβ while time-course assays confirmed that this was rapid, occurring within 2 min, and sustained for at least 30 min for both TP isoforms (Fig. 1A and B). RhoA activation through GPCRs predominantly occurs by coupling to G_12_ (Gα_12_/Gα_13_) members but may also occur through Gq coupling, in certain settings at least [31–33]. Herein, over-expression of dominant negative forms of Gα_12_ (Gα_12_^G228A^), but not of Gαq (Gαq^Q209 l,D277N^), significantly impaired U46619-mediated RhoA activation through both TPα (*p* = 0.0011 and *p* = 0.8011 respectively) and TPβ (*p* = 0.0043 and *p* = 0.9235 respectively; Fig. 1C).

To extend these studies, we also examined U46619-mediated stress fibre formation in HEK.TPα and HEK.TPβ cells by monitoring F-actin polymerization and Rho-dependent phosphorylation, and inactivation, of the actin depolymerizing agent cofilin using *anti*-phospho-cofilin antibodies directed to phosphoSer^3^ [34]. Throughout the latter, assays were normalised for total cofilin expression as presented in the lower panels in each of the figures. Whilst the control HEK 293 cells failed to show any changes in stress-fibre formation in response to U46619 (1 nM–10 μM), both TPα and TPβ induced rapid and profound F-actin polymerization with optimal responses occurring using 10 nM U46619 (Fig. 2A). Moreover, U46619 induced rapid and concentration-dependent cofilin phosphorylation in both HEK.TPα and HEK.TPβ cells with optimal responses occurring with 1 μM U46619 by both TP isoforms (Fig. 2B) while there was no cofilin phosphorylation in either cell line in response to the drug vehicle or in HEK 293 cells in response to U46619 stimulation (Fig. 2B). Furthermore, this U46619-mediated phosphorylation of cofilin was inhibited by over-expression of the dominant negative form of Gα_12_ (Gα_12_^G228A^), while the dominant negative form of Gαq (Gαq^Q209l,D277N^) had no significant effect (Fig. 2C). The Rho kinase inhibitor Y27632 (10 μM) effectively abolished U46619-induced cofilin phosphorylation by both TPα and TPβ (data not shown).

Collectively, these data confirm that both TPα and TPβ can independently couple to both Gq-dependent PLCβ activation to mobilize Ca^2+^ from IP_3_-operated intracellular stores, for example, and to G_12_-dependent RhoA activation and effector coupling leading to cofilin phosphorylation and inactivation and to F-actin polymerization.

### 3.2 The effect of prostacyclin/cAMP and NO/cGMP on TPα- and TPβ-mediated PLCβ- and RhoA-signaling

Amongst the many functional differences identified thus far between the individual TPα and TPβ isoforms [22], one of the most significant relates to the differential heterologous desensitization of their signaling by the vasodilatory autocoids prostacyclin [21], prostaglandin (PG) D_2_ [27] and nitric oxide [20]. Hence, in view of those differential sensitivities of Gq/PLCβ-mediated signaling by TPα and TPβ to both prostacyclin/cAMP and NO/cGMP [20,21], coupled to the well documented inhibitory actions of cAMP and cGMP on Rho-mediated signaling in response to various agents including TXA_2_ and thrombin, such as within platelets and vascular smooth muscle [35,36], we next investigated the effects of prostacyclin and NO on RhoA-mediated signaling by the individual TPα and TPβ isoforms.

**Fig. 3:**
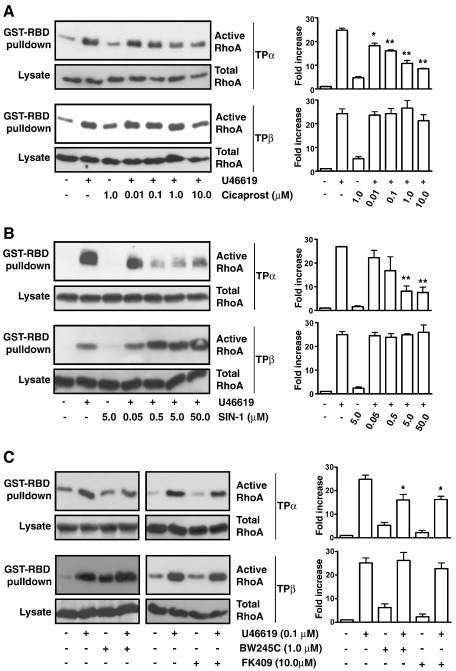
Cicaprost- and SIN-1-induced desensitization of TP-mediated signaling. Panels A–C: HEK.TPα and HEK.TPβ cells were serum starved for 5 h before treatment for 10 min with vehicle (Panels A and B), 0.01–10 μM Cicaprost (Panel A), 0.05–50 μM SIN-1 (Panel B), 1 μM BW345C or 10 μM FK409 (Panel C). Thereafter, cells were incubated with 100 nM U46619 for 10 min (Panels A–C). Active Rho was precipitated from the cell lysates using GST-RBD fusion protein, separated by SDS-PAGE and immunoblotted with an *anti*-RhoA antibody (Upper panels). Cell lysates were analyzed by western blotting for total RhoA expression (Lower panels). The bar charts to the right of the panels signify mean fold increases in Rho activation ± S.E.M. (*n* = 3–6) where basal levels were assigned a value of 1.0. The asterisks indicates that the level of U46619-mediated RhoA activation was significantly reduced in the presence of Cicaprost, Sin-1, BW245C and FK409 where ⁎ and ⁎⁎ indicates *p* < 0.05 and *p* < 0.01, respectively.

**Fig. 4:**
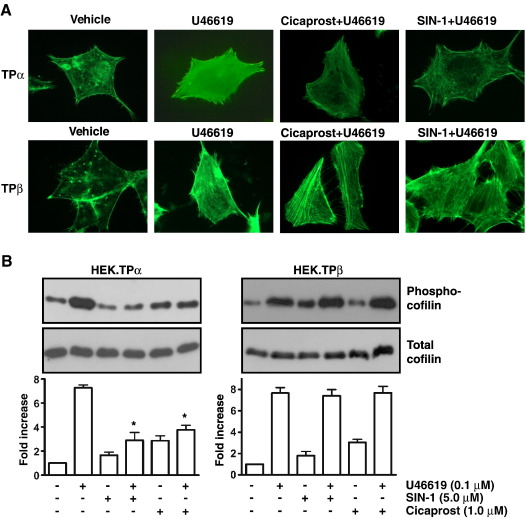
Cicaprost- and SIN-1-induced desensitization of TP-mediated signaling. Panel A: HEK.TPα and HEK.TPβ cells were serum starved for 5 h before treatment for 10 min with vehicle (Vehicle), 10 nM U46619 (U46619), 100 nM Cicaprost followed by 10 nM U46619 (U46619, Cicaprost) or 500 nM SIN-1 followed by 10 nM U46619 (U46619, SIN-1). F-actin formation was detected with Alexa Fluor® 488 phalloidin followed by fluorescence microscopy. Images presented are representative of the majority of cells examined and of 3/4 independent experiments. Panel B. HEK.TPα and HEK.TPβ cells were serum starved for 5 h before treatment for 10 min with vehicle (−), 1 μM Cicaprost or 10 μM SIN-1 (Panel B). Thereafter, cells were incubated with vehicle (−) or 100 nM U46619 for 10 min. Cell lysates were separated by SDS-PAGE and immunoblotted with *anti*-Phospho cofilin (Upper panels) or *anti*-cofilin (Lower panels) antibodies. The bar charts signify mean fold increases in cofilin phosphorylation ± S.E.M. (*n* = 3–6) where basal levels were assigned a value of 1.0. The asterisks indicates that the level of U46619-mediated cofilin phosphorylation was significantly reduced in the presence of Cicaprost or Sin-1 where ⁎ indicates *p* < 0.05. (The reader is referred to the web version of this article to see color images of this figure, where relevant.)

Initially the effect of the prostacyclin analogue Cicaprost (1 μM) or the NO-donor SIN-1 (5 μM) on U46619-mediated [Ca^2+^]_*i*_ mobilization and RhoA activation and signaling by TPα and TPβ was examined. Consistent with our previous findings [20,21], pre-incubation with either Cicaprost or SIN-1 significantly impaired U46619-induced [Ca^2+^]_*i*_ mobilization by TPα but had no effect on signaling by TPβ (Fig. 1; Supplemental Data). While Cicaprost did not induce substantial RhoA activation *per se* in HEK.TPα, HEK.TPβ or HEK 293 cells (data not shown), it significantly impaired U46619-induced RhoA activation by TPα expressed in HEK.TPα cells in a concentration-dependent manner (Fig. 3A). On the other hand, Cicaprost had no effect on RhoA activation by TPβ, even at 10 μM Cicaprost (Fig. 3A). Similarly, SIN-1 also significantly impaired U46619-mediated RhoA activation by TPα in a concentration-dependent manner but had no effect on RhoA activation by TPβ, even at 50 μM SIN-1 (Fig. 3B). While Cicaprost (1–10 μM) and SIN-1 (5–50 μM) each significantly impaired U46619-induced F-actin polymerization by both TPα and TPβ, consistent with the inhibitory effects of cAMP/PKA and cGMP/PKG on both the Ca^2+^-dependent and Ca^2+^-independent paths, it was apparent that at lower concentrations both Cicaprost (100 nM) and SIN-1 (500 nM) impaired F-actin polymerization in HEK.TPα cells but neither agent affected such responses in HEK.TPβ cells (Fig. 4A). Moreover, U46619-induced cofilin phosphorylation by TPα was also significantly impaired by either Cicaprost or SIN-1, while neither agent affected such responses in HEK.TPβ cells (Fig. 4B), regardless of concentration. Consistent with the latter data, the PGD_2_ analogue BW245C and the alternative NO donor FK409 also significantly impaired U46619-mediated RhoA activation (Fig. 3C) and cofilin phosphorylation (data not shown) by TPα but had no effect on signaling by TPβ (Fig. 3C and data not shown).

**Fig. 5:**
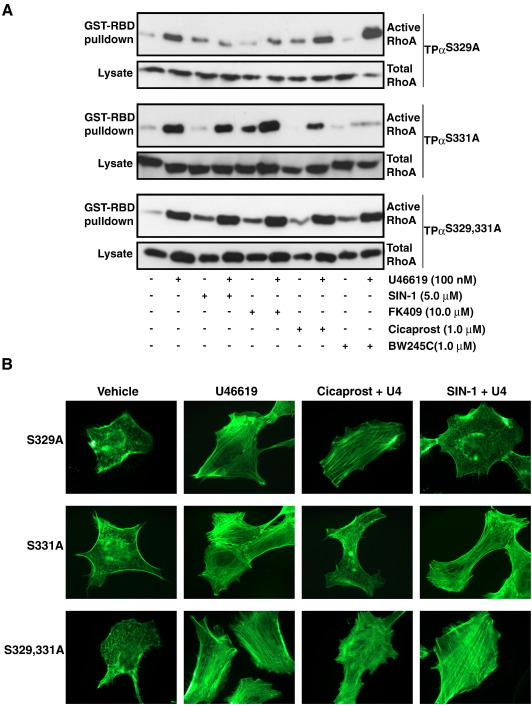
Cicaprost- and SIN-1-induced desensitization of TP Signaling in HEK 293 cells. Panel A: HEK.TPα^S329A^, HEK.TPα^S331A^ and HEK.TPα^S331,329A^ cells were serum-starved for 5 h before treatment for 10 min with vehicle (−), 5 μM SIN-1, 10 μM FK409, 1 μM Cicaprost or 1 μM BW245C as indicated. Thereafter, cells were incubated for 10 min with vehicle (−) or 100 nM U46619 for 10 min. Active Rho was precipitated from the cell lysates using GST-RBD fusion protein, separated by SDS-PAGE and immunoblotted with an *anti*-RhoA antibody (Upper panels). Cell lysates were analyzed by western blotting for total RhoA expression (Lower panels). Panel B: HEK.TPα^S329A^, HEK.TPα^S331A^ and HEK.TPα^S331,329A^ cells were serum starved for 5 h before treatment for 10 min with vehicle, 500 nM SIN-1 or 100 nM Cicaprost. Thereafter, cells were incubated for 10 min with vehicle (−) or 10 nM U46619 for 10 min. F-actin formation was detected with Alexa Fluor® 488 phalloidin followed by fluorescence microscopy. Images presented are representative of the majority of cells examined in 8 independent fields and of 3/4 independent experiments. (The reader is referred to the web version of this article to see color images of this figure, where relevant.)

We have previously established that while both prostacyclin analogues, such as Cicaprost, and NO-donors, such as SIN-1, were indeed capable of cross-desensitizing or impairing Gq/PLCβ signaling by TPα, they did so by entirely independent mechanisms and at different, though adjacent, sites. Specifically, prostacyclin-desensitization occurs by direct PKA phosphorylation of Ser^329^ while NO-desensitization occurs through PKG phosphorylation of Ser^331^, both within the unique C-tail domain of TPα [20,21]. Hence, to further investigate the mechanism by which SIN-1 and Cicaprost impair signaling by TPα, we examined their effects on U46619-induced [Ca^2+^]_*i*_ mobilization, Rho activation, F-actin polymerization and cofilin phosphorylation by TPα and its specific site directed variants TPα^S329A^, TPα^S331A^, TPα^S329,331A^ defective in the Cicaprost-sensitive PKA (at Ser^329^), NO-sensitive PKG (at Ser^331^) or both (at Ser^329,331^) phosphorylation sites, as previously described by us [20,21]. Consistent with those previous studies, pre-incubation with SIN-1 specifically impaired U46619-induced [Ca^2+^]_*i*_ mobilization in HEK.TPα and HEK.TPα^S329A^ cells while having no affect on such signaling in HEK.TPα^S331A^ and HEK.TPα^S329,331A^ cells (Fig. 2; Supplemental Data). Moreover, both SIN-1 and the alternative NO-donor FK409 also specifically impaired U46619-induced RhoA activation by TPα and TPα^S329A^ cells but had no affect on signaling by TPα^S331A^ and TPα^S329,331A^ (Fig. 5A). Additionally, SIN-1 and FK409 also impaired U46619-induced F-actin polymerization, at low agonist concentration, and cofilin phosphorylation by TPα (Fig. 4 and data not shown) and TPα^S329A^ cells, but had no affect on signaling by TPα^S331A^ and TPα^S329,331A^ (Fig. 5B and data not shown). On the other hand, pre-stimulation with Cicaprost impaired U46619-induced [Ca^2+^]_*i*_ mobilization and RhoA activation by TPα and TPα^S331A^ while having no affect on signaling by TPα^S329A^ and TPα^S329,331A^ (Fig. 2; Supplemental Data and Fig. 5A). Consistent with this, the PGD_2_ receptor (DP) agonist BW245C also impaired RhoA activation by TPα (Fig. 3C) and TPα^S331A^ without affecting signaling by TPα^S329A^ and TPα^S329,331A^ (Fig. 5A). Additionally, Cicaprost specifically impaired U46619-induced F-actin polymerization and cofilin phosphorylation by TPα (Fig. 4) and TPα^S331A^ cells but had no affect on signaling by TPα^S329A^ and TPα^S329,331A^ (Fig. 5B and data not shown).

Taken together these data clearly indicate that both Gq/PLCβ-mediated [Ca^2+^]_*i*_ mobilization and the G_12_/RhoGEF-dependent RhoA activation and cofilin phosphorylation by TPα are specifically impaired by the potent vasodilators SIN-1 and Cicaprost. On the other hand, neither agonist-induced Gq/PLCβ nor G_12_/RhoA signaling by TPβ is directly affected by either vasodilator. Moreover, consistent with our previous findings [20,21], our data herein further suggest that the mechanisms whereby Cicaprost and SIN-1 impair both the Gq/PLCβ-mediated [Ca^2+^]_*i*_ pathway and the Rho-dependent pathway are similar but entirely independent where NO/SIN-1-mediated desensitization occurs through a PKG-dependent mechanism involving direct phosphorylation of TPα at Ser^331^ while that of prostacyclin/Cicaprost involves a PKA-dependent mechanism where Ser^329^ is the phospho-target.

### 3.3 TPα- and TPβ-mediated RhoA signaling in primary human AoSMCs

**Fig. 6:**
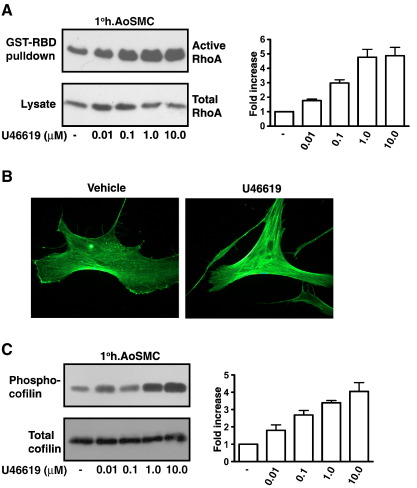
TP-mediated Rho signaling in 1° human Aortic Smooth Muscle Cells. Panel A: 1° AoSMCs were serum starved for 20 h before treatment for 10 min with vehicle (−) or 0.001–10 μM U46619 as indicated. Active Rho was precipitated from the cell lysates using GST-RBD fusion protein, separated by SDS-PAGE and immunoblotted with an *anti*-RhoA antibody (Upper panels). Cell lysates were analyzed by western blotting for total RhoA expression (Lower panels). Panel B: 1° AoSMCs were serum starved for 5 h before treatment for 10 min with vehicle or 1 μM U46619 for 10 min. F-actin formation was detected with Alexa Fluor® 488 phalloidin followed by fluorescence microscopy. Images presented are representative of the majority of cells examined and of 3/4 independent experiments. Panel C: 1° AoSMCs were serum starved for 20 h before treatment for 10 min with vehicle (−) or 0.001–10 μM U46619 as indicated. Cell lysates were separated by SDS-PAGE and immunoblotted with *anti*-Phospho cofilin (Upper panels) or *anti*-cofilin (Lower panels) antibodies to detect phosphorylated and total cofilin expression. The bar charts to the right of the panels signify mean fold increases in Rho activation or cofilin phosphorylation ± S.E.M. (*n* = 3–6) where levels of basal levels are assigned a value of 1.0. (The reader is referred to the web version of this article to see color images of this figure, where relevant.)

**Fig. 7:**
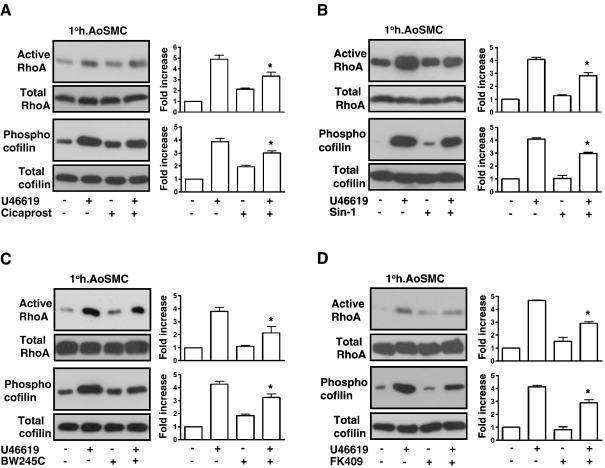
Desensitization of TP Signaling in 1° h.AoSMCs. 1° AoSMCs were serum starved for 20 h before treatment for 10 min with vehicle (−), 1 μM Cicaprost (Panel A), 5 μM SIN-1 (Panel B), 1 μM BW245C (Panel C) or 10 μM FK409 (Panel D). Thereafter, cells were incubated for 10 min with vehicle (−) or 1 μM U46619, as indicated. Active Rho was precipitated from the cell lysates using GST-RBD fusion protein, separated by SDS-PAGE and immunoblotted with an *anti*-RhoA antibody (GST-RBD pulldown) while aliquots of cell lysates were analyzed by western blotting for total RhoA expression, phospho-cofilin and total cofilin expression as indicated. The bar charts to the right of the panels signify mean fold increases in Rho activation or cofilin phosphorylation ± S.E.M. (*n* = 3–6) where basal levels are assigned a value of 1.0. The asterisks indicates that the level of U46619-mediated RhoA activation and cofilin phosphorylation was significantly reduced in the presence of Cicaprost, Sin-1, BW245C or FK409 where ⁎ indicates *p* < 0.05.

We next investigated TP-mediated Rho activation and cytoskeletal signaling in a physiologically relevant, vaso-responsive model by investigating U46619-induced signaling in 1° h.AoSMCs, cells that express both TPα and TPβ [30]. Consistent with our findings herein in HEK.TPα and HEK.TPβ cells, stimulation of 1° h.AoSMCs with U46619 led to rapid RhoA activation with maximal responses observed with 100–1000 nM U46619 (Fig. 6A). Stimulation of 1° h.AoSMCs also led to rapid F-actin polymerization (Fig. 6B) and cofilin phosphorylation with optimal responses generated using 1 μM U46619 (Fig. 6C). While the IP agonist Cicaprost did not lead to substantial RhoA activation and cofilin phosphorylation relative to the drug vehicle *per se*, it significantly impaired such U46619-mediated signaling in 1° h.AoSMCs (Fig. 7A). Consistent with this, the specific PGD_2_ receptor (DP) agonist BW245C also significantly impaired RhoA activation (Fig. 7C) and cofilin phosphorylation in 1° h.AoSMCs. Similarly, while the NO donors SIN-1 and FK409 alone did not induce substantial RhoA signaling relative to the drug vehicle *per se*, they each significantly impaired U46619-induced RhoA activation and cofilin phosphorylation following their pre-incubation in 1° h.AoSMCs (Fig. 7B and D). Moreover, while Cicaprost, Sin-1, BW245C or FK409 did not induce F-actin polymerization *per se*, they each significantly impaired U46619-induced F-actin polymerization (data not shown).

Hence, taken together, both NO-donors and prostanoid vasodilatory agents, such as prostacyclin and PGD_2_ signaling through the prostacyclin (IP) and PGD_2_ (DP) receptors, respectively, can impair U46619-mediated Rho activation and cytoskeletal signaling in 1° h.AoSMCs. Moreover, our data generated in the HEK.TPα and HEK.TPβ cell lines over-expressing the individual TPα and TPβ isoforms, respectively, suggest that such inhibitory responses of prostacyclin and NO are mediated, at least in part, at the interface of the stimulatory GPCR (i.e. the TP). More specifically, by directly targeting TPα, prostacyclin and NO may impair its RhoA-signaling both at the level of TPα itself in addition to at the level of the well documented targets of cAMP/PKA and cGMP/PKG [35,36]. On the other hand, as TPβ is not as such a direct target of prostacyclin- or NO-mediated phosphorylation and inhibition, their effect on TPβ-mediated RhoA signaling may be solely manifest at a later point in the cascade [36]. Clarity on this issue in 1° h.AoSMCs is, however, confounded by the fact that h.AoSMCs express both TPα and TPβ isoforms [30] and, therefore, it is possible that the vasodilatory agents NO and Cicaprost may target TPα, TPβ or both in addition to other downstream targets.

**Fig. 8:**
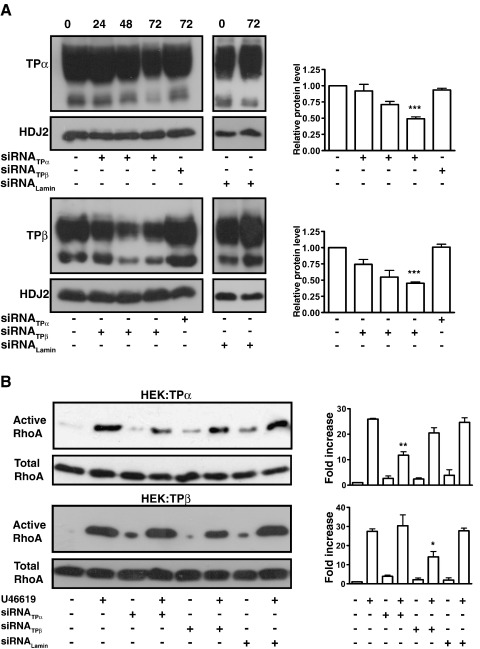
Effect of siRNA-mediated down-regulation of TPα and TPβ Expression on Rho-signaling in HEK 293 cells. Panel A: HEK.TPα and HEK.TPβ cells were transfected with siRNA-directed to TPα (siRNA_TPα_) and TPβ (siRNA_TPβ_), respectively, for 0–72 h. As controls, HEK.TPα cells were transfected with siRNA_TPβ_ or HEK.TPβ cells were transfected with siRNA_TPα_ for 72 h. Alternatively, as additional controls, HEK.TPα cells or HEK.TPβ cells were transfected with siRNA_LaminA/C_ for 72 h (Right panels). HA-tagged TPα or TPβ expression was detected by immunoblotting using *anti*-HA 3F10 antibody (Upper panels) or equal protein loading was verified by secondary screening of blots with an *anti*-HDJ2 antibody (Lower panels). Panel B: HEK.TPα and HEK.TPβ cells were transfected with siRNA_TPα_, siRNA_TPβ_ or siRNA_LaminA/C_ for 72 h, as indicated. Thereafter, cells were incubated for 10 min with vehicle (−) or 100 nM U46619 for 10 min. Active Rho was precipitated from the cell lysates using GST-RBD fusion protein and immunoblotted with an *anti*-RhoA antibody (Upper panels). Cell lysates were analyzed by western blotting for total RhoA expression (Lower panels). The bar charts to the right of the panels signify mean fold changes in TP isoform expression (A) and Rho activation (B) ± S.E.M. (*n* = 3–6) where basal levels are assigned a value of 1.0. The asterisks indicates that TPα (siRNA_TPα_) and TPβ (siRNA_TPβ_) expression (Panel A) or U46619-mediated RhoA activation (Panel B) was significantly reduced in the presence of their respective siRNAs where ⁎, ⁎⁎ and ⁎⁎⁎ indicates *p* < 0.05, *p* < 0.01 and *p* < 0.001 respectively.

To address this issue, we generated small interfering RNA (siRNA)-targeting agents to selectively disrupt or knock-down expression of TPα and TPβ in 1° h.AoSMCs. To begin with, the siRNA agents were validated by examining their ability to affect TPα and TPβ expression and RhoA signaling in HEK.TPα and HEK.TPβ cells. Under optimized experimental conditions, we observed effective isoform-specific knock-down of both TPα and TPβ expression following 72 h incubation of HEK.TPα and HEK.TPβ cells with siRNA_TPα_ and siRNA_TPβ_, respectively (Fig. 8A), with ~ 50–60% specific knock-down achieved as assessed by densitometry and radioligand binding assay in each case (Fig. 8A and data not shown). On the other hand, the siRNA_TPα_ did not affect TPβ expression in HEK.TPβ cells and siRNA_TPβ_ did not affect TPα expression in HEK.TPα cells (Fig. 8A) thereby confirming the specificity of the TPα and TPβ isoform-directed siRNAs. Additionally, RNA_*i*_ directed to Lamin A/C, acting as a control, had no effect on either TPα or TPβ expression in either cell line (Fig. 8A). Moreover, pre-incubation of HEK.TPα cells with siRNA_TPα_ significantly impaired U46619-induced RhoA activation but had no significant effect on such signaling in HEK.TPβ cells (Fig. 8B). Conversely the *anti*-TPβ siRNA_TPβ_ significantly impaired U46619-induced RhoA activation in HEK.TPβ cells but had no effect on such signaling in HEK.TPα cells (Fig. 8B). RNA_*i*_ directed to Lamin A/C had no effect on either TPα- or TPβ-mediated RhoA activation (Fig. 8B). Consistent with these findings, siRNAs directed to TPα and TPβ also impaired U46619-mediated F-actin polymerization and cofilin phosphorylation in HEK.TPα and HEK.TPβ cells, respectively, and in an entirely isoform specific manner (data not shown).

**Fig. 9:**
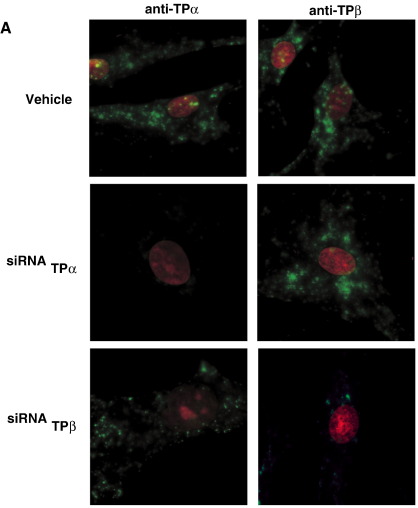

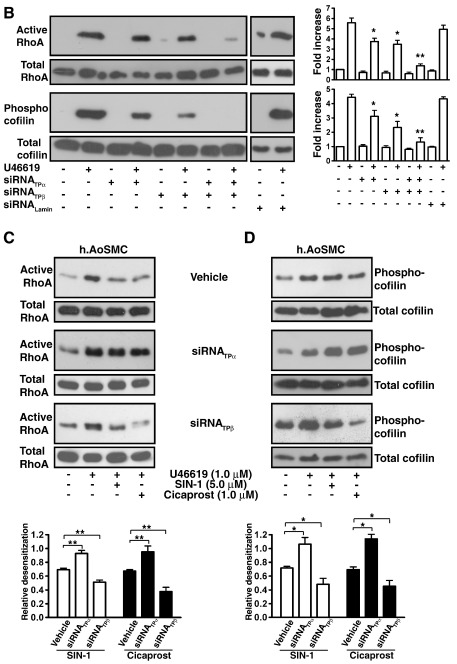
Effect of siRNA-mediated down-regulation of TPα and TPβ Expression on Rho-signaling in 1° h.AoSMCs. Panels A–D: 1° AoSMCs were transfected with siRNA-directed to TPα (siRNA_TPα_), TPβ (siRNA_TPβ_) or Lamin A/C (siRNA_Lamin A/C_) for 72 h where non-transfected cells served as controls, as indicated. In Panel A, following fixation and permeabilization, cells were screened by indirect immunoflourescence microscopy with *anti*-TPα or *anti*-TPβ isoform specific 1° antibody and stained using FITC-labelled goat *anti*-rabbit IgG, where cell nuclei were counter stained with propidium iodide. In Panel A, data are representative of 3 independent experiments where a total of 16 independent fields of cells were analysed for each TP isoform. It was estimated that there was greater than 70% reduction TPα/TPβ expression in 80% of cells analysed. In Panel B, cells were serum-starved for 20 h before treatment for 10 min with vehicle or 1 μM U46619 as indicated. Active Rho was precipitated from the cell lysates using GST-RBD fusion protein, separated by SDS-PAGE and immunoblotted with an *anti*-RhoA antibody (Active RhoA) while cell lysates were analyzed for total RhoA expression, phospho-cofilin and total cofilin expression as indicated. Panels C and D, the cells were serum-starved for 20 h before treatment for 10 min with vehicle, 1.0 μM Cicaprost or 5.0 μM SIN-1. Thereafter, cells were incubated for 10 min with vehicle (−) or 1 μM U46619. In Panel C, active Rho was precipitated from the cell lysates using GST-RBD fusion protein, separated by SDS-PAGE and immunoblotted with an *anti*-RhoA antibody (Upper panels) while cell lysates were analyzed for total RhoA expression (Lower panels). In Panel D, cell lysates were immunoblotted with *anti*-Phospho cofilin (Upper panels) or *anti*-cofilin (Lower panels) antibodies. In Panel B, the bar charts to the right signify mean fold changes in Rho activation and cofilin phosphorylation ± S.E.M. (*n* = 3–6) where basal levels are assigned a value of 1.0. The asterisks indicates that U46619-mediated RhoA activation and cofilin phosphorylation was significantly reduced in the presence of their respective TPα (siRNA_TPα_) and TPβ (siRNA_TPβ_) siRNAs where ⁎ indicates *p* < 0.05 and *p* < 0.01, respectively. The bar charts below Panels C and D depict mean reductions (± S.E.M., *n* = 3–6) in U46619-mediated Rho activation (C) and cofilin phosphorylation (D) in response to pre-treatment with Sin-1 and Cicaprost, and the asterisks indicate that the level of desensitization was significantly altered in the presence of the siRNA_TPα_ or siRNA_TPβ_ where ⁎ and ⁎⁎ indicates *p* < 0.05 and *p* < 0.01, respectively. (The reader is referred to the web version of this article to see color images of this figure, where relevant.)

Having established the specificity of the siRNA reagents to impair expression and RhoA-dependent signaling by both TPα and TPβ in HEK 293 cells, we next examined their ability to affect expression and signaling by the individual TP isoforms in h.AoSMCs. The effective delivery and utility of the latter siRNAs in 1° h.AoSMCs was initially confirmed whereby the siRNA_TPα_ impaired expression of TPα but not of TPβ while siRNA_TPβ_ reduced expression of TPβ but not of TPα (Fig. 9A). Consistent with the reduced expression of TPα and TPβ following incubation of the 1° h.AoSMCs with the isoform-specific siRNAs, there were reductions in U46619-induced Rho activation and cofilin phosphorylation in the presence of RNA_*i*_ directed to either TP but not directed to Lamin A/C (Fig. 9B). Moreover, incubation of the 1° h.AoSMCs with both siRNA_TPα_ and siRNA_TPβ_ led to a further significant reduction in U46619-induced Rho activation and cofilin phosphorylation. Hence, these data clearly suggest that both TPα and TPβ contribute to the Rho activation in h.AoSMCs.

We next examined the effect of the inhibitory vasodilatory agents SIN-1 and Cicaprost on U46619-mediated RhoA activation and signaling in 1° h.AoSMCs in the presence of the respective TP-isoform specific siRNA reagents. In the absence of siRNA, SIN-1 and Cicaprost significantly impaired U46619-mediated RhoA activation (Fig. 9C) consistent with our earlier findings in both 1° h.AoSMCs (Fig. 7A and B) and in HEK.TPα cells (Fig. 3B and C). Following 72 h incubation with siRNA_TPβ_, the NO donor SIN-1 specifically impaired U46619-mediated RhoA activation in h.AoSMCs to levels greater than that observed in vehicle treated cells. On the other hand, the inhibitory effect of SIN-1 on U46619-mediated RhoA activation in 1° h.AoSMCs was significantly less in the presence of siRNA_TPα_ at 72 h (Fig. 9C). Similarly, SIN-1 impaired U46619-mediated cofilin phosphorylation in the presence of siRNA_TPβ_ to levels similar to those observed in vehicle-treated cells but its ability to impair U46619-signaling in the presence of siRNA_TPα_ was almost fully abolished (Fig. 9D). Moreover, the prostacyclin analogue Cicaprost significantly impaired Rho activation (Fig. 9C) and cofilin phosphorylation (Fig. 9D) in 1° h.AoSMCs pre-treated with siRNA_TPβ_ to levels similar to that observed in the control, vehicle-treated cells while its ability to impair that signaling in cells pre-treated with the siRNA_TPα_ was almost completely abolished.

Hence, we conclude that, similar to that which occurs for TP-mediated Gq/PLCβ activation, both the NO and prostacyclin analogues SIN-1 and Cicaprost impair TP-mediated cytoskeletal changes involving RhoA activation and cofilin phosphorylation in 1° h.AoSMCs and that they do so, at least in part, by specifically and directly targeting TPα, impairing its downstream signaling. On the other hand, neither vasodilatory agent directly target TPβ. Hence, TPα- and TPβ-mediated RhoA signaling functionally diverge at the point at which prostacyclin and NO affect the RhoA signaling cascade.

## 4 Discussion

Much of the molecular events that underlie the complex physiologic processes of platelet aggregation and thrombosis, VSM contraction, migration, proliferation involve the fundamental reorganization of the cellular cytoskeleton [5]. A key step in this cytoskeletal reorganization involves alterations in MLC phosphorylation that occurs either through Ca^2+^-dependent activation of MLCK or through the alternative Ca^2+^-independent pathway, involving RhoA [1,2,5,6]. For example, the processes that contribute to platelet activation and secretion and shape change are under the dual control of the Gq*/*Ca^2+^-dependent and G_12_/Ca^2+^-independent pathways, respectively [1,8].

The prostanoid TXA_2_ plays an essential role within the vasculature inducing a range of cellular responses including platelet shape change and aggregation; contraction of vascular and bronchial smooth muscle (SM) cells; mitogenic and hypertrophic growth of VSM cells; inhibition of angiogenesis/vascularization [37–39]. Elevations in the levels of TXA_2_, its synthase or its receptor have been implicated in various cardiovascular disorders including thrombosis, myocardial infarction, unstable angina, atherosclerosis, systemic- and pregnancy-induced hypertension and ischemic heart disease, processes in which RhoA dysfunction is widely implicated [7]. In humans, TXA_2_ signals through 2 distinct isoforms referred to as TPα and TPβ [13,15,22]. While the functional requirement for two types of receptor for TXA_2_ in humans is unknown there is substantial evidence that they may have distinct physiologic/pathophysiologic roles [16,17,19,22].

Bearing this in mind and the growing appreciation of the critical role of the RhoA-mediated Ca^2+^-independent pathways to both normal and disease-processes within the vasculature [2,5,6], the central aim of the current study was to investigate the ability of the individual TPα and TPβ isoforms to regulate RhoA signaling. Moreover, in view of the critical involvement of inhibitory agents including prostacyclin and NO, that largely signal through cAMP and cGMP second messengers, in regulating RhoA-dependent mechanisms [1,6,35,36] coupled to their role in differentially regulating TPα and TPβ-mediated Gq/PLCβ signaling [20,21,27], we also sought to investigate the impact of both vasodilators on RhoA signaling through the individual TP isoforms.

To this end, we investigated the ability of TPα and TPβ to mediate RhoA signaling in established clonal HEK 293 cell lines that over-express the individual TP isoforms [20,21] and in cultured 1° AoSMCs, a physiologically relevant cell type that expresses both TPα and TPβ [26,30]. Throughout these studies, TP-mediated RhoA signaling was determined by monitoring its activation-dependent interaction with the Rho-binding domain (RBD) of its effector rhotekin in GST-RBD pulldown assays in response to the TXA_2_ mimetic U46619. Moreover, we also investigated the ability of TPα and TPβ to regulate events downstream of RhoA: effector coupling by monitoring U46619-induced F-actin polymerization and cofilin phosphorylation. The ubiquitously expressed actin-depolymerising factor cofilin readily undergoes Rho/Rho kinase-dependent phosphorylation at Ser^3^ either by the LIM kinase 1/2 [3] and was used herein as a monitor of events downstream of Rho kinase in the Rho signaling cascade. Our conclusions are several-fold. Both TPα and TPβ expressed in HEK 293 cells readily induced RhoA activation, F-actin polymerization and cofilin phosphorylation in response to U46619. In general, GPCR-mediated RhoA activation largely occurs through a G_12_, mainly Gα_13_, -dependent mechanism but in certain settings, particularly at higher agonist concentrations, may also occur through a Gq-mechanism through the specific involvement of the LARG (Leukemia-associated Rho guanine-nucleotide exchange factor), but not the p115- or PDZ-, member of the RGS-containing RhoGEF family [1,31–33]. Hence, herein, we sought to clarify the involvement of G_12_ and Gq on TP-mediated RhoA signaling and found that dominant negative forms of Gα_12_ (Gα_12_^G228A^), not of Gαq (Gαq^Q209l,D277N^), significantly impaired U46619-mediated RhoA activation and cofilin phosphorylation. Collectively, these data confirmed that both TPα and TPβ can independently couple to Gq/PLCβ activation and Gα_12_/Gα_13_/RhoGEF-RhoA activation and are in agreement with a host of studies in mouse platelets whereby the single TP in that species couples to Gq/PLCβ and to G_12_/RhoA activation to independently regulate platelet activation (aggregation and secretion) and platelet shape change responses, respectively [1,8].

Thereafter, we investigated the effect of the selective prostacyclin analogue Cicaprost and the NO donor SIN-1 on TP-mediated RhoA activation and signaling. Consistent with our previous reports [20,21,27], Gq/PLCβ-mediated [Ca^2+^]_*i*_ mobilization by TPα, but not by TPβ, was desensitized in response to both Cicaprost and NO stimulation. In keeping with this, TPα-mediated RhoA activation, F-actin polymerization and cofilin phosphorylation was also specifically impaired by Cicaprost and SIN-1 while neither agent affected Rho-mediated signaling by TPβ. As stated, while both prostacyclin and NO desensitize TPα-mediated Gq/PLCβ signaling, they do so by entirely independent mechanisms involving direct PKA- and PKG- mediated phosphorylation of TPα at Ser^329^ and Ser^331^, respectively, within its unique C-tail domain [20,21]. Hence, we next compared the effect of SIN-1 and Cicaprost on Rho-signaling by TPα^S329A^, TPα^S331A^, TPα^S329,331A^, variants of TPα defective in the prostacyclin-sensitive PKA (at Ser^329^), NO-sensitive PKG (at Ser^331^) or both (at Ser^329,331^) phospho-target sites [20,21]. While SIN-1 and the alternative NO-donor FK409 impaired U46619-induced RhoA activation, cofilin phosphorylation, F-actin polymerization as well as [Ca^2+^]_*i*_ mobilization by TPα and TPα^S329A^, they had no affect on signaling by TPα^S331A^ and TPα^S329,331A^. Conversely, both Cicaprost and the PGD_2_ receptor agonist BW245C impaired RhoA activation, cofilin phosphorylation, F-actin polymerization and [Ca^2+^]_*i*_ mobilization by TPα and TPα^S331A^ but had no affect on signaling by TPα^S329A^ and TPα^S329,331A^. Collectively, these data suggest that both Gq/PLCβ-mediated [Ca^2+^]_*i*_ mobilization and G_12_/RhoGEF -dependent RhoA activation of its effector rhotekin and cofilin phosphorylation by TPα, but not by TPβ, are specifically impaired by the potent vasodilators SIN-1 and Cicaprost in this cellular context, at least. Of course the inhibitory effects of both prostacyclin and NO, and other agents that signal through cAMP and cGMP, on RhoA signaling are widely documented and form an essential component of the homeostatic regulatory mechanism that determines the balance between activation and inhibition, particularly within the vasculature [1,6,36]. Hence, it is arguable that the effects of Cicaprost and SIN-1 on TPα-mediated Rho signaling in HEK 293 cells are perhaps somewhat predictable. However, the fact that RhoA-mediated signaling by TPα^S331A^ and TPα^S329,331A^ is unaffected by SIN-1 while that signaling by TPα^S329A^ and TPα^S329,331A^ is unaffected by Cicaprost clearly suggest that the observed effects of SIN-1 and Cicaprost on TPα, in the HEK 293 over-expression system at least, are due to direct effects on TPα itself, namely through site specific prostacyclin-induced PKA (at Ser^329^) and NO-induced PKG (at Ser^331^) phosphorylation rather than at some other intermediary in the RhoA signaling cascade. Moreover, in keeping with that hypothesis, the finding that agonist-induced G_12_/RhoA signaling by TPβ is not affected by either prostacyclin or NO again suggests that the effects of both vasodilators are due to direct effects on TPα itself and is entirely consistent with previous findings involving both prostacyclin- and NO-mediated desensitization of TPα and TPβ signaling through the Gq/PLCβ effector system [20,21]. The fact that we do not observe any measurable inhibitory effects on TPβ-mediated RhoA signaling by either Cicaprost or SIN-1, such as might be expected to occur at a later point in the signaling cascade [36], could in theory be due to the fact that the level of TP receptor expression in the HEK 293 stable cell lines produces an overriding forward signal, overwhelming any inhibitory effects of prostacyclin or NO.

Therefore, we extended our studies by investigating TP-mediated Rho activation and cytoskeletal signaling in the more physiologically relevant primary human aortic smooth muscle cells. As expected, stimulation of cultured 1° h.AoSMCs with U46619 led to a concentration-dependent RhoA activation, F-actin polymerization and cofilin phosphorylation. Moreover, Cicaprost (IP agonist) and BW245C (DP agonist) and the NO donors SIN-1 and FK409 each significantly impaired such U46619-induced RhoA activation, F-actin polymerization and cofilin phosphorylation in 1° h.AoSMCs. Hence, collectively, both NO-donors and the vasodilatory prostanoids prostacyclin and PGD_2_ readily desensitize TP-mediated Rho activation and cytoskeletal signaling in 1° h.AoSMCs, findings entirely predicted from and in keeping with outcomes from other systems [6,8,35,36]. However, as stated, our data generated in the HEK 293 cell lines over-expressing the individual TPα and TPβ isoforms suggest that such inhibitory responses of prostacyclin and NO are mediated, at least in part, directly at the level of TPα itself rather than at the level of other well documented targets of cAMP/PKA and cGMP/PKG on Rho signaling [35,36]. Human AoSMCs express both TPα and TPβ isoforms [26,30]. Hence, through the use of TP isoform-specific siRNA, we sought to determine whether TPα and TPβ independently contribute to U46619-induced RhoA activation and signaling in 1° h.AoSMCs and to ascertain whether the inhibitory effects of NO and/or Cicaprost may directly target TPα, or indeed TPβ, at the level of the receptor itself and/or in addition to other downstream targets [36]. Under optimized experimental conditions, the specificity and utility of the siRNA_TPα_ and siRNA_TPβ_ reagents were validated whereby we observed effective isoform-specific knock-down of both TPα and TPβ expression and RhoA-mediated signaling in their respective HEK 293 cell lines. Moreover, the effective delivery and utility of the latter siRNAs in 1° h.AoSMCs was confirmed whereby the siRNA_TPα_ reduced expression of TPα but not of TPβ, while siRNA_TPβ_ reduced expression of TPβ but not of TPα. It was notable that the level of siRNA-mediated impairment of TPα and TPβ expression in the 1° h.AoSMCs was significantly higher than observed in HEK.TPα or HEK.TPβ cells. The reason for this apparent discrepancy is simply owing to the fact that the stably transfected HEK 293 cell lines express TPα and TPβ in abundance (~ 2 pmol/mg protein) relative to that expressed in 1° h.AoSMCs (20–50 fmol/mg protein) and hence, the inability of the siRNA to completely konckdown TPα or TPβ expression in HEK.TPα or HEK.TPβ cells was not surprising. Consistent with their reduced expression in the 1° h.AoSMCs, there was a significant reduction in U46619-mediated Rho activation and cofilin phosphorylation in the presence of RNA_*i*_ directed to either TPα or TPβ but not to Lamin A/C confirming that both TPα and TPβ contribute to the RhoA activation in h.AoSMCs. While SIN-1 and Cicaprost significantly impaired U46619-mediated RhoA activation in the presence of the siRNA directed to Lamin A/C to levels similar to that in vehicle-treated cells, the inhibitory action of both agents on RhoA activation and cofilin phosphorylation in 1° h.AoSMCs exposed to the siRNA_TPα_ was substantially impaired. On the other hand, in the presence of siRNA_TPβ_ both SIN-1 and Cicaprost reduced U46619-mediated RhoA, F-actin polymerization (data not shown) and cofilin phosphorylation to levels not significantly different to those observed in vehicle-treated cells. Hence, both the NO and prostacyclin impair TP-mediated cytoskeletal changes involving RhoA activation, F-actin polymerization and cofilin phosphorylation in 1° h.AoSMCs and they do so, at least in part, by specifically and directly targeting TPα impairing its downstream signaling. On the other hand, neither vasodilatory agent directly target TPβ.

As stated, it is widely held that agents that signal through either cAMP- or cGMP-second messenger systems play a critical counter-balancing/inhibitory affect on RhoA-mediated signaling cascades [35,36] as well as regulating Rho-mediated transcriptional responses through the serum response factor [40]. In fact within the vasculature, there is a critical reciprocal relationship between RhoA signaling and expression and that of NO-signaling and expression of endothelial nitric oxide synthase (eNOS) [6,36,41,42]. Moreover, in platelets there is a differential effect whereby cAMP/PKA inhibits both the Gq/PLCβ-mediated aggregation and secretion and the G_12_/Rho-mediated shape change while cGMP/PKG signaling inhibits the former Gq/Ca^2+^ dependent mechanism but does not affect the latter RhoA/Ca^2+^ independent mechanism [35]. Clearly many of the actions of cAMP and cGMP on RhoA signaling are mediated through their respective second messenger kinases PKA and PKG, respectively [6,35,36,43] and more recently it has been established that this may largely occur through their direct phosphorylation of RhoA itself at an identical site, namely Ser^188^ within its hypervariable region [36,42,44,45]. Whilst phosphorylation of RhoA at Ser^188^ does not apparently alter its association with either RhoGEFs or RhoGAPs (GTPase activating proteins), it significantly increases its interaction with RhoGDI (GDP dissociation inhibitor) thereby reducing the level of membrane bound RhoA and impairing its ability to activate its key effectors including Rho Kinases [36,46]. Moreover, in a recent study investigating NGF-mediated RhoA responses in neuronal PC12 cells, Nusser et al. provided *in vitro* and *in vivo* evidence to suggest that Ser^188^ phosphorylation of RhoA impairs activation of Rho kinase (ROCK 1/2), but does not affect its ability to activate other Rho effectors including rhotekin, mDia-1 and PKN [47]. From their studies, they proposed that Ser^188^ phosphorylation of RhoA may act as a ‘secondary molecular switch’ capable of overriding GTP-elicited activation of certain RhoA effectors, such as ROCK, but directing it to signal with (an)other subset of Rho effectors, perhaps in a cell specific manner. Returning to studies herein on TPα- and TPβ-mediated RhoA signalling, both NO and prostacyclin directly target RhoA phosphorylation at Ser^188^ through their regulation of PKG and PKA signaling, respectively (data not shown). Hence, RhoA-mediated signaling by TPα is subject to regulation by both direct prostacyclin/PKA and NO/PKG-inhibition mediated through their respective phosphorylation of Ser^329^ and Ser^331^ within the unique C-tail domain of TPα in addition to the more general type of regulation through Ser^188^ phosphorylation of RhoA. On the other hand, TPβ is not a direct target for either PKA or PKG phosphorylation or inhibition, but its RhoA-mediated signaling would be sensitive to RhoA phosphorylation by either second messenger kinase. Whilst it has not as yet been established whether the “molecular switch mechanism” resulting from RhoA^S188^ phosphorylation proposed by Nusser et al. [47] to exist in neuronal cells can be extended to other cell/tissue types, such as smooth muscle, it is tempting to speculate.

**Fig. 10:**
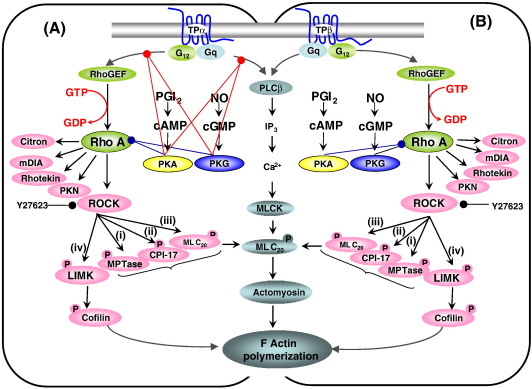
Model of TPα and TPβ-mediated RhoA activation and Signaling. Panels A and B: Agonist (TXA_2_/U46619)-activated TPα and TPβ couples to Gα_q_/PLCβ, yielding increases in IP_3_, mobilization of [Ca^2+^]_*i*_ leading to sequential Ca^2+^/calmodulin-dependent activation of MLCK, MLC_20_ phosphorylation and actomyosin formation resulting in Ca^2+^-dependent F actin polymerization. Agonist-activated TPα and TPβ can simultaneously co-couple to G_12_/RhoGEF to activate RhoA and a host of its effectors including Rho kinase (ROCK), mammalian diaphanous protein (mDIA), Rhotekin, protein kinase (PK)N, amongst many others. ROCK phosphorylates: (i) the myosin-binding subunit (MBS) of myosin phosphatase (MPTase), inhibiting its activity; (ii) and activates CPI-17, a phosphorylation-dependent inhibitor of MPTase; (iii) MLC_20_ itself; (iv) LIM kinase (LIMK) which, in turn, phosphorylates and inactivates the actin depolymerizing agent Cofilin. These combined actions of ROCK contributes to the Rho A/Ca^2+^-independent mechanism for regulating stress fibre formation in non-muscle cells, smooth muscle contraction and platelet shape change. Panel A: The second messenger kinases cAMP-dependent PKA and cGMP-dependent PKG, activated in response to prostacyclin and NO signalling, respectively, cannot only impair TPα-mediated Gq-PLCβ signaling and G_12_-RhoGEF signaling through direct phosphorylation of TPα at Ser^329^ and Ser^331^, respectively, but may also impair that TPα-mediated signalling, such as at the level of RhoA itself through Ser^188^ phosphorylation. Panel B: On the other hand, as TPβ is not subject to direct PKA or PKG phosphorylation, its signaling by prostacyclin or NO may only be regulated at downstream intermediary level(s), such as at the level of RhoA (Ser^188^) phosphorylation. Y27632 is a selective inhibitor of Rho kinase. (The reader is referred to the web version of this article to see color images of this figure, where relevant.)

Hence, as presented in our model (Fig. 10), we propose that as TPα is directly targeted for inhibition by prostacyclin and NO, its signaling would be fully impaired by either vasodilator beginning at the level of the receptor itself. On the other hand, as TPβ is not subject to direct PKA or PKG phosphorylation, its signaling by prostacyclin or NO may only be regulated at downstream intermediary level(s), such as at the level of RhoA phosphorylation. In the event that the ‘phospho-RhoA^Ser188^ switch mechanism’ exists within TXA_2_-responsive VSM or indeed in platelets, RhoA signaling through TPβ may be directed away from one effector system, such as ROCK signaling, in the direction of another effector(s), such as rhotekin, mDia-1 and PKN (Fig. 10) as proposed by Nusser et al. [47] in the neuronal system, or indeed toward other subset(s) of the many diverse RhoA effectors, perhaps in a cell specific manner. Final clarification as to whether such a mechanism exists will require further detailed investigation.

Hence, in summary TPα- and TPβ-mediated RhoA signaling functionally diverge at the point at which prostacyclin and NO affect the RhoA signaling cascade. These data further support the hypothesis that TPα is the major regulatory TP isoform involved in vascular hemostasis being a direct target for inhibition of both its Gq/PLCβ/Ca^2+^-dependent and G_12_/RhoA/Ca^2+^-independent signaling by prostacyclin and NO within the vasculature. On the other hand, as TPβ remains unaffected by either agent, at the interface of the receptor at least, the functional role of TPβ remains to be further clearly defined. The data herein highlight further critical differences between the TPα and TPβ receptor isoforms in terms of their regulation of Rho signaling that are likely to be physiologically relevant in human tissues such as SM and suggest that selective targeting and impairment of TPα-mediated signaling may offer a useful therapeutic approach in the treatment of certain vascular diseases such as systemic- and pulmonary-hypertension in which both TXA_2_ and RhoA dysfunction has been implicated [2,48,49]. Moreover, the data also suggests that for effective impairment of TPβ-mediated RhoA activation and signaling in such clinical settings, it may be necessary to fully antagonize it at the level of the TPβ receptor itself rather than at a later downstream step, such as most typically at the level Rho kinase/ROCK inhibition [2].

## Figures and Tables

**Figure 1:**
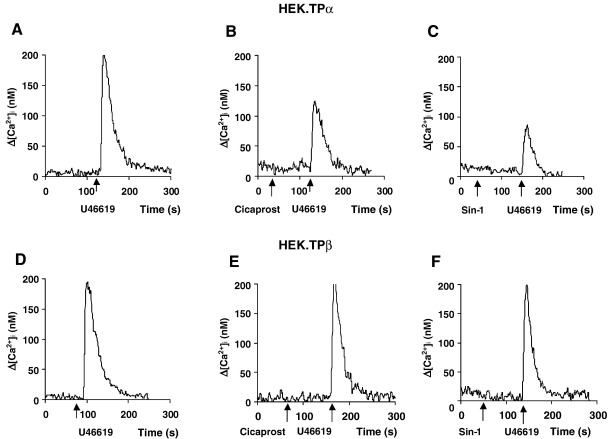
Cicaprost- and SIN-1-induced Desesitization of TP-mediated [Ca^2+^]*i* Mobilization. HEK.TPα (i-iii) and HEK.TPβ (iv-vi) cells, transiently co-transfected with pCMV:Gαq and preloaded with Fura2/AM, were stimulated with 1 μM U46619 (i and iv) or 1 μM Cicaprost followed by 1 μM U46619 (ii and v) or 10 μM SIN-1 followed by 1 μM U46619 (iii and vi), where ligands were added at the times indicated by arrows. Data presented are representative of at least four independent experiments and are plotted as changes in [Ca^2+^]i (Δ[Ca^2+^]i (nM)) as a function of time (s) following ligand stimulation. Data are plotted as Δ[Ca^2+^]i (means ± S.E.M; n=4). Actual mean changes in U46619-mediated [Ca^2+^]i (nM ± S.E.) were as follows: i, Δ[Ca^2+^]i = 188.1 ± 6.4 nM; ii, Δ[Ca^2+^]i = 132 ± 12.7 nM; iii, Δ[Ca^2+^]i = 87.4 ± 12.5 nM; iv, Δ[Ca^2+^]i = 191.1 ± 16.3 nM; v, Δ[Ca^2+^]i = 212 ± 16.2 nM; vi, Δ[Ca^2+^]i = 167.5 ± 11.3 nM.

**Figure 2:**
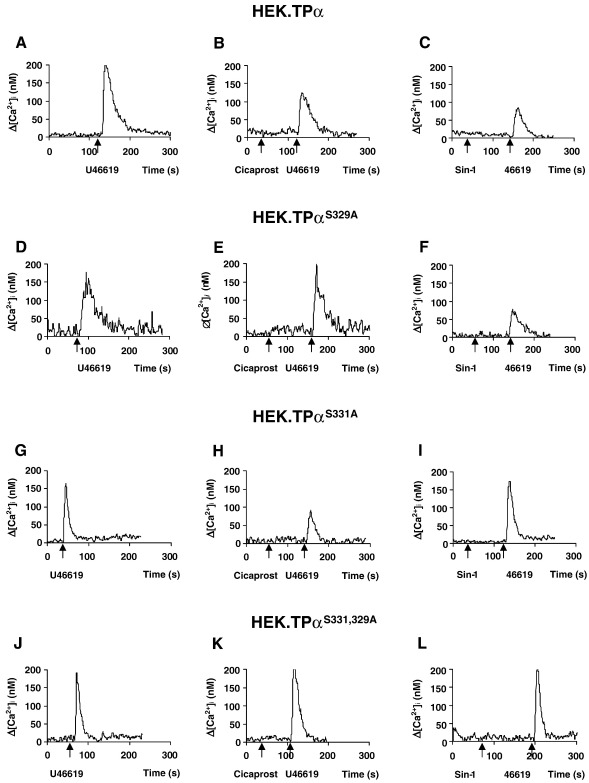
Cicaprost- and SIN-1- Desensitization of TP-induced [Ca^2+^]*i* Mobilization in HEK 293 cells. Panel A: HEK.TPα (i-iii), HEK.TPα^S329A^ (iv-vi), HEK.TPα^S331A^ (vii-ix) and HEK.TPα^S331,329A^ (x –xii) cells, transiently co-transfected with pCMV:Gαq and preloaded with Fura2/AM, were stimulated with either 1 μM U46619 (i, iv, vii, x), 1 μM Cicaprost followed by 1 μM U46619 (ii, v, vii, xi ) or 10 μM SIN-1 followed by 1 μM U46619 (iii, vi, viii, xii) where ligands were added at the times indicated by arrows. Data presented are representative of at least four independent experiments, and are plotted as changes in intracellular Ca^2+^ (Δ[Ca^2+^]i (nM)) as a function of time (s) following ligand stimulation. Data are plotted as Δ[Ca^2+^]i (means ± S.E.M; n=4). Actual mean changes in U46619-mediated Δ[Ca^2+^]i (nM ± S.E.) were as follows: i, Δ[Ca^2+^]i = 188.1 ± 6.4 nM; ii, Δ[Ca^2+^]i = 132 ± 12.7 nM; iii, Δ[Ca^2+^]i = 87.4 ± 12.5 nM; iv, Δ[Ca^2+^]i = 155.6 ± 4.4 nM; v, Δ[Ca^2+^]i = 146 ± 19.3 nM; vi, Δ[Ca^2+^]i = 77 ± 20.2 nM; vii, Δ[Ca^2+^]i = 201 ± 9.1 nM; viii, Δ[Ca^2+^]i = 58.2 ± 18.4 nM; ix, Δ[Ca^2+^]i = 181 ± 19.6 nM; x, Δ[Ca^2+^]i = 198.4 ± 14.3 nM; xi, Δ[Ca^2+^]i = 164.1 ± 15.6 nM; xii, Δ[Ca^2+^]i = 193.6 ± 23.2 nM

**Fig. 1:**
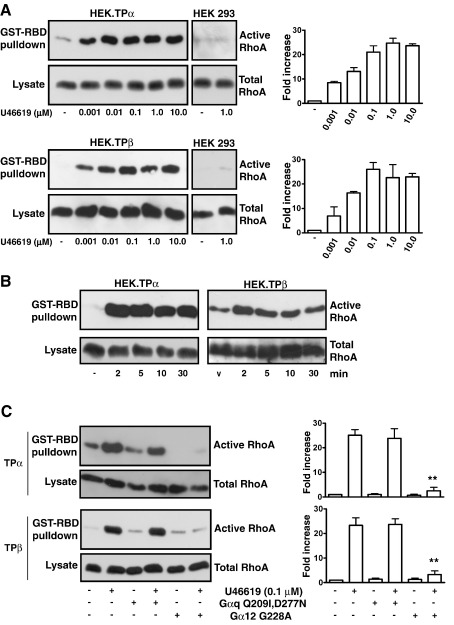
TPα- and TPβ-mediated RhoA Activation. HEK.TPα, HEK.TPβ and HEK293 cells were serum-starved for 5 h before treatment (Panel A) for 10 min with vehicle or the indicated concentrations of U46619 or (Panel B) with 100 nM U46619 for the specified times, where cells treated with vehicle for 30 min acted as the control. Panel C: Alternatively, HEK.TPα and HEK.TPβ cells were transiently transfected with plasmids encoding Gαq^Q209l,D277N^ or Gα_12_^G228A^ . Some 48 h post-transfection, cells were serum-starved for 5 h before treatment for 10 min with vehicle or 100 nM U46619. Active Rho was precipitated from the cell lysates using the Rho pulldown assay involving its binding to the GST-RBD (Rho binding domain of rhotekin)-fusion protein, separated by SDS-PAGE and immunoblotted with *anti*-RhoA antibody (Upper panels). Aliquots of cell lysates (typically 10 μl /lane corresponding to 1.25% of total cell lysate) were also analyzed for total RhoA expression with *anti*-RhoA antibody (Lower panels). The bar charts to the right of the panels signify mean fold increases in Rho activation ± S.E.M. (*n* = 3–6) where basal levels are assigned a value of 1.0.

**Fig. 2:**
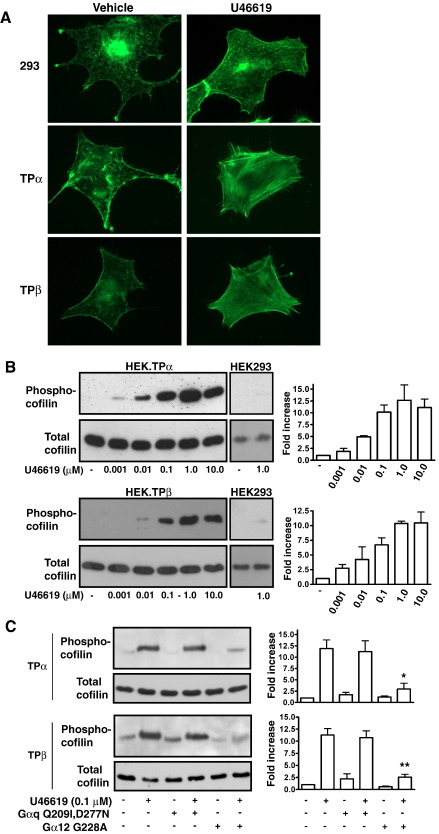
TPα and TPβ-mediated F-Actin Polymerization and Cofilin Phosphorylation. Panel A: HEK.TPα, HEK.TPβ and HEK293 cells were serum-starved for 5 h before treatment with the vehicle (MEM) or 10 nM U46619 for 10 min. Following fixation and permeabilization, F-actin polymerization was detected with Alexa Fluor® 488 phalloidin followed by fluorescence microscopy. Panel B: HEK.TPα, HEK.TPβ and HEK293 cells were serum-starved for 5 h before treatment for 10 min with 0–10 μM U46619. Panel C: Alternatively, HEK.TPα and HEK.TPβ cells were transiently transfected with plasmids encoding Gαq^Q209l,D277N^ or Gα_12_^G228A^. Some 48 h post-transfection, cells were serum-starved for 5 h before treatment for 10 min with vehicle or 100 nM U46619. Aliquots of the cell lysates (typically 10 μl/lane corresponding to 1.25% of total cell lysate) in B and C were separated by SDS-PAGE and immunoblotted with *anti*-Phospho cofilin (Upper panels) or *anti*-cofilin (Lower panels) antibodies to detect phosphorylated and total cofilin expression. The bar charts to the right of the panels signify mean fold increases in cofilin phosphorylation ± S.E.M. (*n* = 3–6) where basal levels were assigned a value of 1.0. (The reader is referred to the web version of this article to see color images of this figure, where relevant.)
